# Incidence and risk factors of neonatal hypoglycemia at Hawassa University Comprehensive Specialized Hospital

**DOI:** 10.1371/journal.pone.0332495

**Published:** 2025-09-15

**Authors:** Sintayehu Amare Tessema, Desalegn Dawit Assele, Henok Bekele Kebede, Yitayew Ewnetu Mohammed

**Affiliations:** 1 Department of Nursing, College of Medicine and Health Sciences, Hawassa University, Hawassa, Ethiopia; 2 Department of Public Health, College of Medicine and Health Sciences, Hawassa University, Hawassa, Ethiopia; 3 Public Health Department, Pharma College Hawassa Campus, Hawassa, Sidama, Ethiopia; 4 Department of Internal Medicine, College of Medicine and Health Sciences, Hawassa University, Hawassa, Ethiopia; KI: Karolinska Institutet, SWEDEN

## Abstract

**Background:**

Neonatal hypoglycemia is the most common metabolic emergency in neonates, with a reported incidence of 15% among neonates overall and 50% among high-risk newborns. If neonatal hypoglycemia is not diagnosed and managed promptly and properly, it can result in brain damage, neurological problems, and death. Over one-third of hypoglycemic neonates die in resource-limited settings. This study aimed to assess the incidence and risk factors of neonatal hypoglycemia in the neonatal intensive care unit at Hawassa University Comprehensive Specialized Hospital, Sidama Region, Ethiopia.

**Methods:**

A retrospective cohort study was conducted among 308 neonates admitted to Hawassa University Comprehensive Specialized Hospital from July 2023 to July 2024. Data were extracted using a standard abstraction format from medical records. Descriptive statistics were summarized using tables and graphs. The Kaplan-Meier survival function and log-rank were used to show a hypoglycemia-free survival rate. An adjusted hazard ratio (aHR) with 95% Confidence interval was used to measure the strength of the association, and the statistical significance was declared at a p-value of less than or equal to 0.05. The Cox proportional hazard regression model assumption was checked by using the Schoenfeld residual test.

**Results:**

The study found that the incidence rate of neonatal hypoglycemia was 3.1 (95% CI: 2.3–4.2) per 100 neonatal days of observation. Female gender (aHR: 3.4; 95% CI: 1.68, 6.83), neonatal sepsis (aHR: 2.1; 95% CI: 1.10–4.00), cesarean section (aHR: 2.1; 95% CI: 1.10, 4.21), preterm (aHR: 5.1; 95% CI: 2.41–10.9), and gestational diabetes mellitus (aHR: 3.4; 95% CI: 1.45, 8.11) were predictors of neonatal hypoglycemia.

**Conclusion:**

The study found that the incidence rate of neonatal hypoglycemia was 3.1 per 100 days. Female sex, preterm birth, neonatal sepsis, cesarean delivery, and maternal gestational diabetes were independently associated with a higher incidence of hypoglycemia. These findings highlight the importance of close monitoring of blood glucose levels in at-risk neonates during neonatal intensive care unit admission.

## Background

Neonatal hypoglycemia (NH) is the most common metabolic disorder in neonates and is associated with brain damage, e.g., posterior and deep grey matter damage, and an increased risk of neurodevelopmental disorders [1–4]. It is characterized by changes in maternal and fetal metabolism and disruptions in insulin production and function [5]. Blood glucose levels of healthy newborns temporarily drop after delivery as a result of the natural adaptation to life outside the womb [6]. Although severe or persistent hypoglycemia is frequently asymptomatic, it can be brought on by an inadequate glucose supply, excessive insulin production, metabolic demand, or malfunctioning counter-regulatory systems [7]. The clinical presentation of NH is usually variable and nonspecific, but the primary clinical symptoms are irritability, shortness of breath, reduced muscle tone, feeding problems, hypothermia, convulsions, or lethargy [8].

NH is a widespread condition in newborns, particularly in low- and middle-income countries (LMICs), and is associated with increased mortality rates in neonatal intensive care units (NICU) [9]. The incidence of NH in newborns ranges from 10% to 20% [10], rises to 50% in high-risk newborns, and continues to rise with additional high-risk factors (infants of diabetic mothers, small for gestational age, those with intrauterine growth restriction, preterm babies, metabolic problems, growth hormone and cortisol disorders, and perinatal stress) [6,11–13]. The burden of NH was high in Africa, ranging from 1.8% in Somalia [14] to 30.5% in Nigeria [15]. In Ethiopia, the occurrence of NH among neonates admitted to the NICU varies by setting; it was 3.81% at Debre Markos Hospital [16] and 25% at Saint Paul’s Hospital, Addis Ababa [17]. Factors such as variations in population characteristics, clinical settings, and management protocols used in institutions may contribute to the significant difference in the prevalence of NH. Studies reported that several factors, such as gestational diabetes mellitus (GDM), advanced maternal age, poor economic conditions, gestational age, sex, hypothermia, birth asphyxia, seizures, low birth weight, small for gestational age, intrauterine growth restriction (IUGR), Sepsis, late initiation of breastfeeding, and tobacco exposure during pregnancy, were related to NH [18–22].

Diagnosis and treatment of neonatal hypoglycemia are challenging due to the absence of specific clinical symptoms. If not recognized and treated promptly, it can result in acute brain injury (e.g., hypoglycemic encephalopathy, seizure), long-term neurologic sequelae (e.g., posterior and deep grey matter damage, neurodevelopmental disorders, cognitive impairment, visual impairment), and death [4,11,23,24]. Early detection is therefore essential, as timely administration of exogenous glucose can minimize the risk of brain injury and mortality [25,26]. The World Health Organization (WHO) has emphasized the importance of preventing, detecting, and treating neonatal hypoglycemia [27]. Nevertheless, significant gaps remain worldwide, as high-resource settings often face a larger number of high-risk neonates, while low and lower-middle-income countries frequently lack access to efficient blood glucose screening, diagnosis, and treatment, which contributes to poor health outcomes [26,28–30]. In Ethiopia, despite efforts to reduce neonatal hypoglycemia-related mortality, hypoglycemia remains the primary cause of NICU admission, with over two-thirds of hypoglycemic neonates dying in the NICU [31].

In Ethiopia, few studies have explored neonatal hypoglycemia and its determinants using a cross-sectional design [17,21]. A previous study found a 6.2% prevalence of NH at Hawassa University Comprehensive Specialized Hospital (HUCSH) NICU, with 35.7% of hypoglycemic neonates dying. However, factors associated with neonatal hypoglycemia were not assessed [32]. There is a lack of cohort data on the true incidence of neonatal hypoglycemia in the NICU and on which factors predict hypoglycemia. Therefore, this study has two primary objectives. First, it assessed the incidence of NH among NICU patients. Second, it examines risk factors associated with the development of NH.

## Methods

### Study period and setting

This study was conducted at Hawassa University Comprehensive Specialized Hospital (HUCSH) from July 2023 to July 2024. The hospital, which has over 10 departments, offers neonatal intensive care to inborn and outborn neonates with various morbidities. The neonatology unit, under the Department of Pediatrics and Child Health, has implemented significant interventions such as continuous positive airway pressure (CPAP) at birth, plastic wrapping of preterm babies, minimal handling, intermittent kangaroo mother care (KMC), NICU resuscitation team coordination, infection control, use of sterile water for CPAP, blood culture sensitivity report for sepsis treatment, cleaning campaign, and active expression of breastfeeding. The level III NICU ensures continuous availability of personnel and equipment for life support. Between July 2023 and July 2024, 786 neonates were admitted to the NICU.

### Study design and population

An institution-based retrospective cohort study was conducted among neonates admitted to the NICU. All neonates admitted to the neonatal intensive care unit (NICU) at Hawassa University Comprehensive Specialized Hospital (HUCSH) were the source population. All neonates admitted to the NICU at HUCSH from July 2023 to July 2024 were the study population. All neonates admitted to the NICU who had a random blood sugar measurement between July 2023 and July 2024 were included in the study. Neonates with incomplete information were excluded from the study.

### Sample size determination and sampling procedure

The sample size was calculated considering the Cox regression model using the following assumption: the probability of the event (the proportion of hypoglycemia of 25%), and the number of neonates who developed hypoglycemia was 49, taken from a study conducted at Saint Paul’s Hospital Millennium Medical College, Addis Ababa, Ethiopia [17]. Thus, the calculated sample size after adding a 15% contingency for missing and incomplete data yields 308. The following STATA command was used:

*power cox, failprob (0.25) wdprob(0.15)*

The sample frame was derived from the NICU registration book, with random numbers generated by STATA 16. A simple random sampling technique was employed to recruit a predetermined sample size from the client registration numbers.

### Variables of the study

The dependent variable of the study was hypoglycemia, defined as a random blood sugar (RBS) level < 45 mg/dl. Independent variables were selected based on previous literature, taking into account the characteristics of the study population and available data on medical records. The independent variables include fetal characteristics: gestational age (calculated from the last menstrual period), 5^th^ minute APGAR score, gender, birth weight, respiratory distress, perinatal asphyxia, sepsis, and meconium aspiration syndrome. Obstetric and medical Factors: parity, gravidity, number of antenatal care visits, mode of delivery (normal vaginal delivery, cesarean section, instrumental delivery), GDM, and preeclampsia.

Operational definitions of variables

**Hypoglycemia**: a plasma glucose level of less than 45 mg/dL [33].

**Preterm** **birth**: live birth before 37 completed weeks of gestational age [34].

**Event**: the occurrence of blood glucose level < 45 mg/dL during the follow-up time (from the date of admission until discharge).

**Censored**: Neonates who were referred to other health institutions or discharged with parental refusal or RBS > 45 mg/dL during discharge were considered censored.

**Perinatal asphyxia** is defined as profound metabolic or mixed acidemia, the persistence of an Apgar score of 0–3 for longer than 5 min, neonatal neurologic sequelae (e.g., seizures, coma, hypotonia, and inability to suck/cry) [35].

**Sepsis** is defined as Clinical signs and symptoms with the presence of risk factors, lab tests (microscopic), or confirmed by blood culture [36].

**Hypothermia**: An abnormal thermal state in which the newborn’s body temperature drops below 36.5°C (97.7°F) [37].

### Data collection tool and procedure

The English version data extraction checklist was designed from related literature [16,17,19,38]. The checklist consists of socio-demographic characteristics, obstetric measurements, and neonatal factors. In HUCSH NICU, blood glucose monitoring follows the Ethiopian Clinical Reference Manual for Advanced Neonatal Care [33]. The NICU primarily admits newborns requiring specialized care and frequent monitoring. All neonates admitted to the NICU will have at least one blood glucose measurement upon admission. For high-risk neonates (preterm, small or large for gestational age, infants of diabetic mothers, neonates with sepsis, or those with feeding problems), blood glucose is checked at admission, then every 3 hours during the first 24 hours, followed by 6–8 hourly intervals depending on clinical status. Data were extracted from neonatal charts (including documented admission and discharge diagnoses and laboratory data) using a data extraction tool for the occurrence of the event. Data were collected by four trained nurses from the medical records of neonates using Kobo Collect.

### Data quality assurance

A pre-test was done on 5% of the study sample neonates’ charts to check for the existence of variables in the registration format on the patient’s chart. Before the data collection period, one day of training was given to data collectors and supervisors. On-site supervision was given to solve any doubts about data collection tools and techniques. The collected information was checked for consistency and completeness on the same day by the principal investigator.

### Data processing and analysis

Data were collected using the ODK Collect version and then exported to STATA version 16 for analysis. Descriptive summaries, including median, proportion, and graphs, were used to describe baseline characteristics. The Kaplan-Meier survival curve and Log-rank test were used to estimate the time to hypoglycemia and to compare the survival curves across baseline categorical variables. The Cox proportional hazard regression model was fitted to identify independent predictors of hypoglycemia. A variable with a p-value < 0.25 in the bivariate analysis was recruited for multivariate analysis to avoid missing potential confounders. An adjusted hazard Ratio (aHR) with 95% CI was used to report the strength of the association. Multi-collinearity was checked using the variance inflation factor, with a mean VIF of 1.14, indicating no collinearity. Variables with a p-value ≤0.05 were considered significant predictors. The necessary Cox proportional hazard regression model assumption was checked by using the Schoenfeld residual test (0.5134).

### Ethical considerations

Ethical clearance was obtained from the Institutional Research Ethics Review (IRER) of the Pharma College, and permission was obtained from the hospital’s administrative bodies. Due to the nature of the retrospective retrieval of patient data, the institutional review board of Pharma College waived the requirement for an informed written consent. The personal informant was kept anonymous throughout the investigation, and information concerning particular personal identifiers, like patient names, was not collected.

## Results

### Neonatal characteristics

A total of 302 neonates were included in the study; six were excluded due to incompleteness. Half of the participants, 151 (50.0%), were male. The vast majority of the participants, 275 (91.1%), were in the first seven days of life. Twelve (4%) participants had very low birth weight (< 1500 gm), 61 (20.2%) had low birth weight (1500–2499 gm), and 92 (30.2%) were preterm. Thirty-nine (12.9%) participants had an APGAR score of less than seven at the fifth minute of life, and 84 (27.8%) had neonatal sepsis. Over a third (34.8%) of neonates had hypothermia, and 70 (23.2%) had received resuscitation (Table 1).

**Table 1:** Neonatal characteristics of neonates admitted to NICU at HUCSH, 2025.

Variables	Categories	Frequency	Percentage
Sex	Male	151	50.0
	Female	151	50.0
Age	≤7 day	275	91.1
	>7 day	27	8.9
Gestational age	Pre-term (<37 weeks)	92	30.5
	Term (37–42 weeks)	210	69.5
Weight in grams	<1500	12	4.0
	1500-2499	61	20.2
	2500-4000	223	73.84
	>4000	6	1.99
Fifth-minute APGAR score	< 7	39	12.9
	≥7-10	263	87.1
Neonatal Sepsis	Yes	84	27.8
	No	218	72.2
RDS	Yes	54	17.9
	No	248	82.1
PNA	Yes	57	18.9
	No	245	81.1
Neonatal jaundice	Yes	30	9.9
	No	272	90.1
Congenital anomaly	Yes	9	3.0
	No	293	97.0
Hypothermia	Absent	197	65.2
	Present	105	34.8
Neonatal Resuscitation	Yes	70	23.2
	No	232	76.8

APGAR: Appearance, Pulse, Grimace, Activity, and Respiration; PNA: Perinatal asphyxia; RDS: Respiratory distress syndrome.

### Obstetric characteristics

Nearly half, 149 (49.3%) of neonate mothers aged between 18 and 24 years, and 165 (54.6%) were born through spontaneous vaginal delivery. About one-fifth (19.9%) of mothers had less than four antenatal care visits, and 30 (9.9%) had hypertensive disorder of pregnancy. Eighteen (6.0%) had a history of abortion, and 60 (19.9%) of mothers had febrile episodes intrapartum (**Table 2**).

**Table 2:** Obstetric-related characteristics of neonates admitted to NICU at HUCSH, 2025.

Variables	Categories	Frequency	Percentage
Age of mothers in years	18-24	149	49.3
	25-30	135	44.7
	>30	18	6.0
Types of birth	Singleton	278	92.1
	Twin	24	7.9
Mode of delivery	SVD	165	54.6
	Assisted VD	3	1.0
	CS	134	44.4
Frequency of ANC	<4	60	19.9
	>=4	242	80.1
Hypertensive disorder of pregnancy	Yes	30	9.9
	No	272	90.1
GDM	Yes	12	4.0
	No	290	96.0
APH	Yes	9	3.0
	No	293	97.0
History of STI	Yes	8	2.6
	No	294	97.4
Intrapartum fever	Yes	60	19.9
	No	242	80.1
History of abortion	Yes	18	6.0
	No	284	94.0
HIV status	Positive	6	2.0
	Negative	296	98.0

ANC: Antenatal care; APH: Antepartum hemorrhage; CS: Cesarean section; GDM: Gestational diabetes mellitus; HIV: Human immunodeficiency virus; STI: Sexually transmitted infections; SVD: Spontaneous vaginal delivery; VD: Vaginal delivery.

### Incidence of hypoglycemia

**Fig 1:**
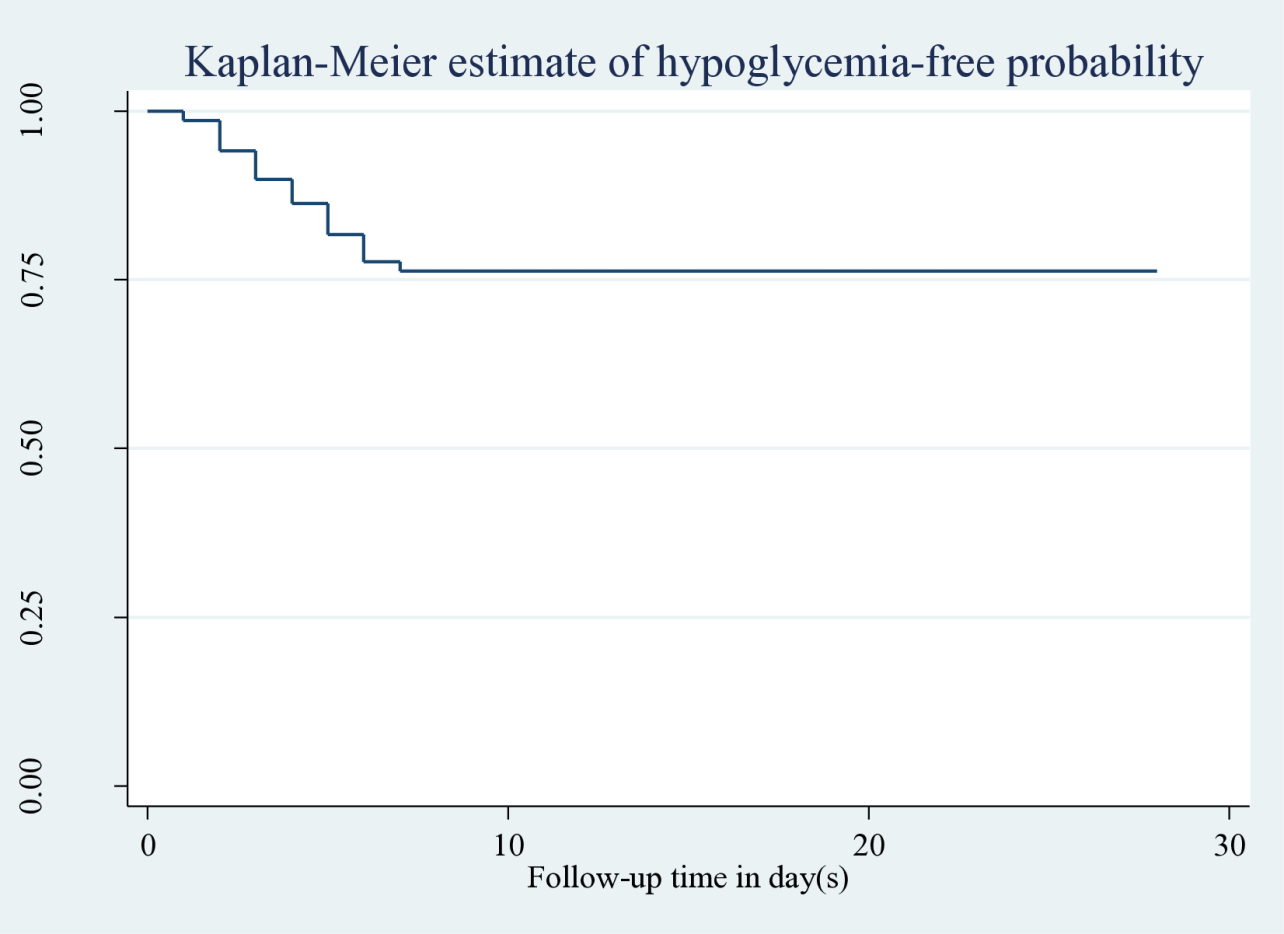
Kaplan-Meier estimate of hypoglycemia-free survival probability in neonates admitted to HUCSH, 2025. Note: Each point on the survival curve corresponds to the probability that a neonate remains free from hypoglycemia up to that specific time.

The follow-up period for 302 neonates was at least one day and up to 28 days, with a median follow-up duration of four days. During the follow-up period, 256 [84.8% (95% CI: 80.6%–88.8%)] were censored, and 46 [15.2% (95% CI: 11.1%–19.3%)] experienced hypoglycemia. The total time at risk was 1455 neonatal days, with an incidence rate of hypoglycemia of 3.1 (95% CI: 2.3–4.2) per 100 neonatal day observations. Hypoglycemia-free probability at the end of the first, third, fifth, and seventh days was 98.6%, 89.1%, 79.7%, and 73.6%, respectively (Fig 1).

**Fig 2:**
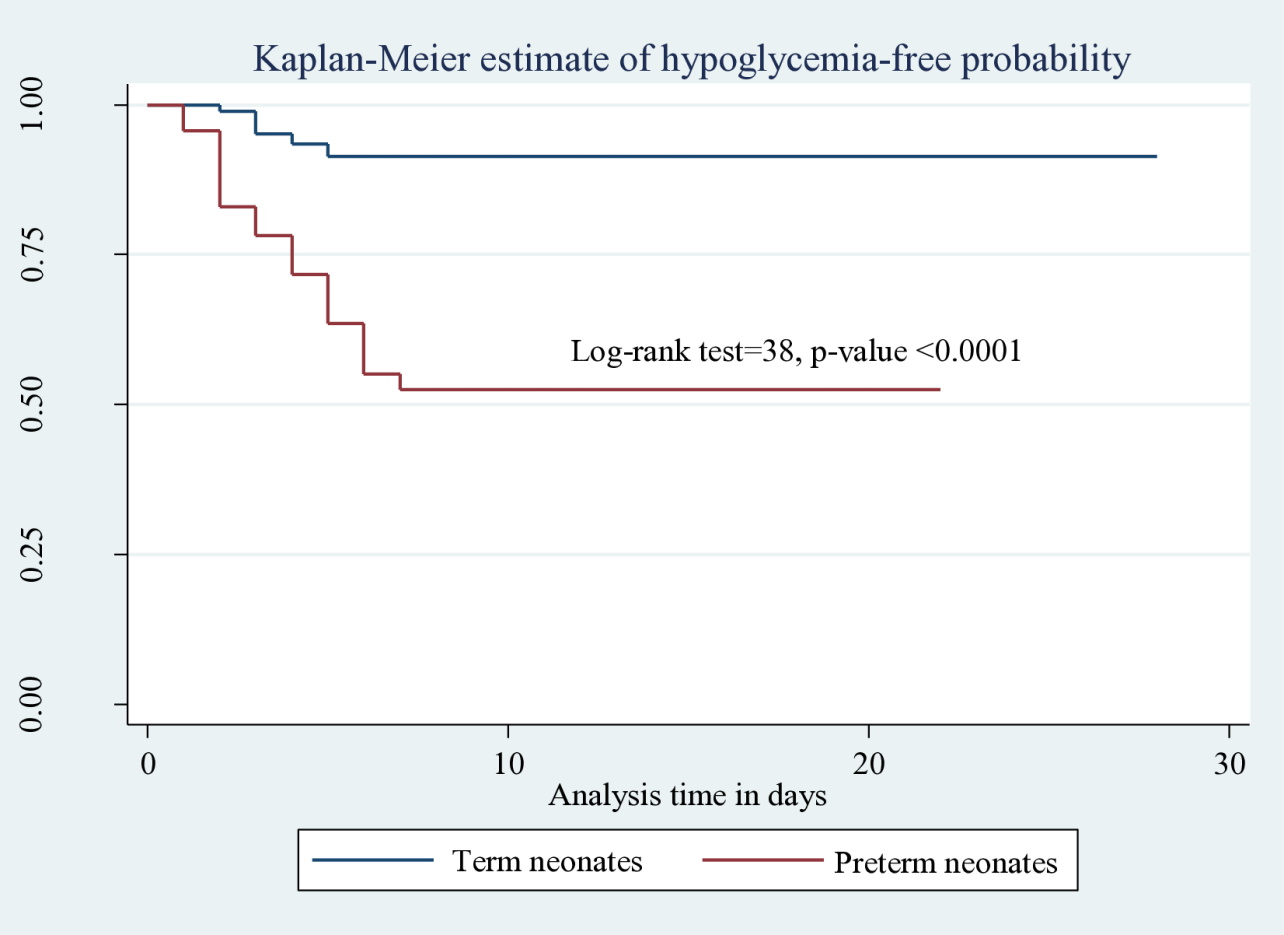
Kaplan-Meier estimate of hypoglycemia-free survival probability based on gestational age of neonates admitted to HUCSH, 2025.

The incidence rate of hypoglycemia among preterm neonates was 7.3 [95% CI: 5.22–10.2] per 100 neonatal per day observation; it was 1.2 [95% CI: 0.6–2.1] per 100 neonatal per day observation for term neonates (log-rank χ2=38, p<0.001) (Fig 2)

**Fig 3:**
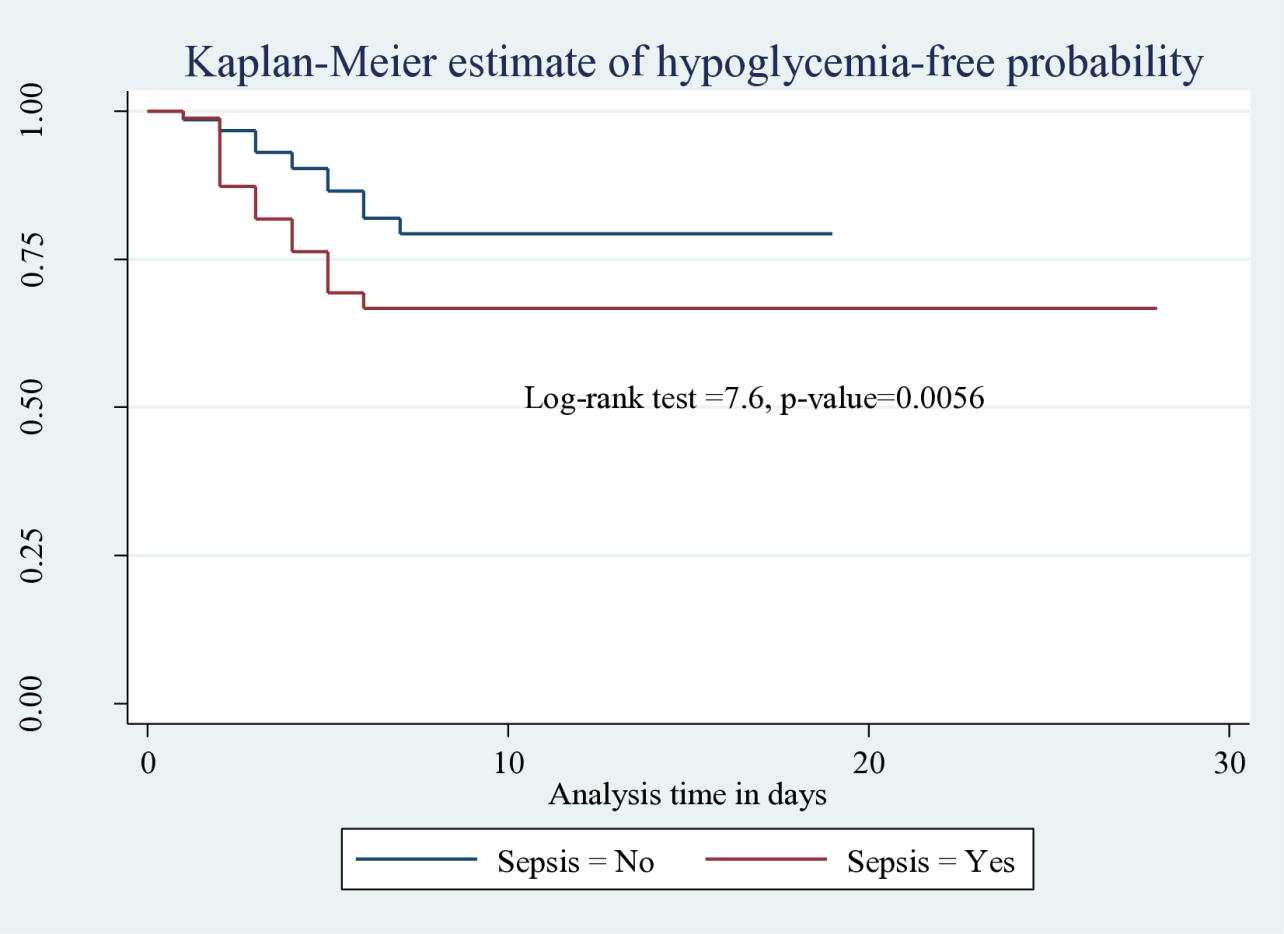
Kaplan-Meier estimate of hypoglycemia-free survival probability based on neonatal sepsis among neonates admitted to HUCSH, 2025.

The incidence rate of hypoglycemia among neonates who had been diagnosed with sepsis was 4.3 [95% CI; 2.82–6.6] per 100 neonatal day observation; it was 2.5 [95% CI; 1.73–3.81] per 100 neonatal day observation for neonates not diagnosed with sepsis (log-rank χ2=7.6, p<0.0056) (Fig 3)

### Predictors of Neonatal Hypoglycemia

In the bivariable Cox-proportional hazard model, sex of neonate, gestational age, birth weight, neonatal sepsis, mode of delivery, hypertensive disorder of pregnancy, mother’s HIV status, and gestational diabetes mellitus were found to have a p-value < 0.25. However, in the final multivariable Cox-proportional hazard model, sex, sepsis, GDM, mode of delivery, and gestational age were predictors of neonatal hypoglycemia.

The hazard ratio of hypoglycemia was 3.4 (aHR: 3.4; 95% CI: 1.68, 6.83) times higher for female neonates when compared to males. The hazard ratio of neonatal hypoglycemia among neonates diagnosed with sepsis was 2.1 (aHR: 2.1; 95% CI: 1.10–4.00) times higher than that of those neonates who had not been diagnosed with neonatal sepsis. The hazard ratio of neonatal hypoglycemia for neonates delivered through cesarean section was 2.1 times (aHR: 2.1; 95% CI: 1.10, 4.21) higher than those delivered through SVD. The hazard ratio of hypoglycemia for preterm neonates was 5.1 (aHR: 5.1; 95% CI: 2.41–10.9) times higher than for term neonates. Further, neonates born to mothers with GDM were 3.4 times (aHR: 3.4; 95% CI: 1.45, 8.11) higher hazard of developing NH than their counterparts (**Table 3**).

**Table 3:** Predictors of neonatal hypoglycemia among neonates admitted to HUCSH, 2025.

Variables	Categories	Event	Censored	cHR (95% CI)	aHR (95% CI)
Sex	Male	12(7.9)	139(92.1)	1	1
	Female	34(22.5)	117(77.5)	2.78(1.44, 5.37)	3.4(1.68, 6.83) **
Gestational age	Pre-term (<37 weeks)	34(36.9)	58(63.0)	6.0(3.11, 11.6)	5.1(2.41,10.9) *
	Term (37–42 weeks)	12(5.7)	198(94.3)	1	
Weight	LBW	21(28.8)	52(71.2)	2.37(1.32, 4.24)	1.1(0.58,2.24)
	Normal	25(10.9)	204(89.1)	1	1
Neonatal Sepsis	Yes	21(25.0)	63(75.0)	2.19(1.22,3.92)	2.1(1.1, 4.00) **
	No	25(11.5)	193(88.5)	1	1
RDS	Yes	9(16.7)	45(83.3)	1.20(0.58,2.49)	
	No	37(14.9)	211(85.1)	1	
PNA	Yes	9(15.8)	48(84.2)	1.04(0.50,2.15)	
	No	37(15.1)	208(84.9)	1	
Neonatal jaundice	Yes	7(23.3)	23(76.7)	1.47(0.65,3.28)	
	No	39(14.3)	233(85.7)	1	
Hypothermia	Absent	26(13.2)	171(86.8)	1	
	Present	20(19.0)	85(89.0)	1.26(0.70,2.26)	
Mode of delivery	CS	30(22.4)	104(77.6)	2.78(1.51,5.12)	2.1(1.1, 4.2) *
	SVD	16(9.5)	152(90.5)	1	1
Mothers HIV status	Positive	3(50.0)	3(50.0)	2.82(0.87, 9.17)	0.97(0.26,3.49)
	Negative	43(14.5)	253(85.5)	1	1
HDP	Yes	9(30.0)	21(70.0)	2.28(1.10, 4.73)	1.49(0.63,3.54)
	No	37(13.6)	235(86.4)	1	1
GDM	Yes	8(66.7)	4(33.3)	4.28(1.99, 9.19)	3.4(1.45, 8.11) **
	No	38(13.1)	252(86.9)	1	1

*Significant at a p-value <0.05 level and ** significant at a p-value <0.001 level; aHR: adjusted hazard ratio; CHR: crude hazard ratio; RDS: respiratory distress syndrome; PNA: perinatal asphyxia; HDP: hypertensive disorder of pregnancy; GDM: gestational diabetes mellitus; CS: Cesarean section; SVD: Spontaneous vaginal delivery; LBW: Low birth weight.

### Model adequacy check

**Fig 4:**
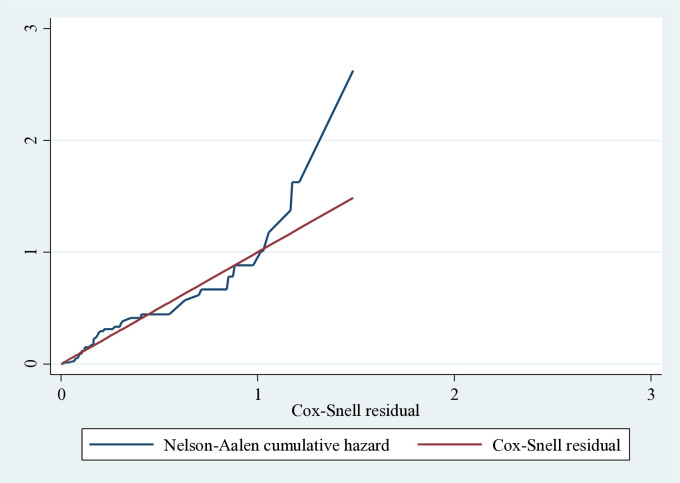
Nelson-Aalen cumulative hazard graph against Cox-Snell residual of neonates admitted to HUCSH, 2025.

The proportional hazard (PH) assumption met the Schoenfeld global test of the entire model (p-value = 0.5134). All covariates met the proportional hazard assumption. The Cox-Snell residuals goodness-of-fit test was used to verify the residuals. It was feasible to determine that the final model fit the data well based on the residual test. The figure below illustrates that the residuals should exhibit a conventional censored exponential distribution with a hazard ratio if the Cox regression model fits the data. The 45° line is closely followed by the hazard function (**Fig 4**).

## Discussion

The study found that the incidence rate of neonatal hypoglycemia was 3.1 (95% CI: 2.3–4.2) per 100 neonatal days of observation. The sex of neonates, neonatal sepsis, GDM, mode of delivery, and gestational age were predictors of neonatal hypoglycemia. The study revealed that 15.2% of neonates experienced neonatal hypoglycemia during the follow-up period. The finding was comparable with a previous study conducted in South East England, 15% [39]. However, the finding was lower than previous studies conducted at Saint Paul’s Hospital Millennium Medical College in Addis Ababa, Ethiopia, which found that 25% [17], Debre Tabor Comprehensive Specialized Hospital 23.6% [21], and at Hiwot Fana Comprehensive Specialized Hospital 21.2% [38]. The discrepancy may be explained by significant variations in the study population and period (some studies included only preterm infants), the prevalence of sepsis, moderate and severe hypothermia, preeclampsia, and GDM.

Similarly, the prevalence of neonatal hypoglycemia was lower than in the study conducted in Italy 25% [40], Croatia (20.7% [22], Poland 38.6% [41], and China (37.4% [42]. This disparity might result from variation in the study population. The study conducted in Croatia included only preterm births [22]; the study in Poland included only early pregnancies complicated by type 1 diabetes [41]; and the study conducted in Italy included only mothers with gestational diabetes mellitus [40].

In our study, female neonates were found to have three times higher hazard of developing neonatal hypoglycemia. However, a clear association between sex and neonatal hypoglycemia has not been reported in previous reports. One study conducted in Croatia, including only premature infants, reported that female sex increases the risk of neonatal hypoglycemia [22]. Potential reasons for the association between female sex and neonatal hypoglycemia could be differences in hormonal and metabolic factors, insulin sensitivity, and differences in feeding patterns.

The study found that newborn sepsis increased the incidence of neonatal hypoglycemia. Neonatal hypoglycemia was 2.1 times greater in neonates diagnosed with sepsis than in neonates who were not diagnosed with sepsis. The finding was supported by a study conducted at public hospitals in the Wolaita Zone [18] and Hiwot Fana Comprehensive Specialized University Hospital, Eastern Ethiopia [38]. In neonates with sepsis, immune cells, muscles, and other organs use more glucose for energy. Glucose consumption increases, especially in cases of severe infection and cytokine storms, increasing the risk of hypoglycemia [43].

The study revealed that cesarean section increases the hazards of neonatal hypoglycemia by 2.1 times compared to spontaneous vaginal delivery. The finding was corroborated by a study conducted in Wolaita Zone [18], Sudan [44], and Iran [45]. This might be explained by the fact that after a cesarean delivery, mothers may feel exhausted and distressed, which can reduce the secretion of breastfeeding hormones [46]. Since cesarean sections were the most common delivery method for mothers with GDM and preeclampsia, which may increase the risk of hypoglycemia, there may not be a causal link between hypoglycemia and cesarean sections.

The gestational age significantly influences neonatal hypoglycemia incidence, with preterm neonates experiencing a 5.1 times higher hazard of hypoglycemia compared to term neonates. The finding was similar to a study conducted at public hospitals of the Wolaita Zone [18] and Hiwot Fana Comprehensive Specialized University Hospital, Eastern Ethiopia [38]. The possible justification for this fact is that immature gluconeogenesis pathways, reduced glycogen and fat stores, inadequate hypoglycemia counter-regulatory responses, lower metabolic reserves, and insufficient substrates are all possible causes of hypoglycemia in preterm neonates [47].

The study found that gestational diabetes mellitus (GDM) is an important risk factor for newborn hypoglycemia. Neonates born to mothers with GDM were at a 3.4 times higher risk of hypoglycemia. The finding is supported by a previous study conducted at Hiwot Fana Comprehensive Specialized University Hospital, Eastern Ethiopia [38] and Wuhan, China [42]. This is explained by the fact that hyperglycemia in the mother can result in excessive glucose consumption by newborns, which may lead to hyperplasia of fetal pancreatic beta cells and an increase in insulin and insulin-like growth factor levels [48].

In our study, a very high percentage of neonates were found to have hypothermia (34.8%). Previous studies have demonstrated that hypothermic neonates are at increased risk of hypoglycemia [17,18,38,49]. However, hypothermia was not identified as a risk factor for neonatal hypoglycemia in our population. In addition, previous studies have reported that low birth weight and perinatal asphyxia are significantly associated with neonatal hypoglycemia [17,18,49]. These factors were not identified as risk factors for neonatal hypoglycemia in our study. Differences in these findings might be due to differences in study population characteristics and clinical settings.

### Strengths and limitations of the study

This study assessed a clinically relevant issue commonly encountered in clinical practice. The findings of the study could shed light on this understudied public health concern and could serve as a baseline for future studies. We used both incidence rate estimation and survival analysis (Kaplan–Meier with log-rank testing, followed by Cox proportional hazards modeling) to strengthen the robustness of our findings. Using survival analysis allowed us to capture both the occurrence and timing of hypoglycemia, providing a more comprehensive understanding than simple prevalence estimates.

However, the study has some limitations. First, due to the retrospective study design and reliance on medical records, some variables were missing, while others were not recorded, which could lead to inadequate assessment of confounding variables that might affect the observed associations. Second, the extracted data, including blood glucose measurements from secondary records, may be subject to information bias during the extraction process. Third, because the study was conducted in a single institution, the findings may not be generalizable to a broader population.

## Conclusion

The study revealed that neonatal hypoglycemia is common among neonates admitted to the NICU, with a prevalence of 15.2% and an incidence rate of 3.1 per 100 days. Factors such as female gender, neonatal sepsis, preterm birth, cesarian delivery, and maternal gestational diabetes were found to have a significant association with neonatal hypoglycemia. Therefore, close monitoring of blood glucose levels is crucial in at-risk neonates admitted to the NICU.

## Supporting information

S1 The dataset analyzed for this study. (XLS)

## Abbreviations

aHR: Adjusted Hazard Ratio
ANC: Antenatal Care
GA: Gestational Age
GDM: Gestational Diabetes Mellitus
IUGR: Intrauterine Growth Retardation
MAS: Meconium Aspiration Syndrome
NICU: Neonatal Intensive Care Unit
